# Risk of Exposure to Eastern Equine Encephalomyelitis Virus Increases with the Density of Northern Cardinals

**DOI:** 10.1371/journal.pone.0057879

**Published:** 2013-02-28

**Authors:** Laura K. Estep, Christopher J. W. McClure, Patrick Vander Kelen, Nathan D. Burkett-Cadena, Stephen Sickerman, José Hernandez, Joseph Jinright, Brenda Hunt, John Lusk, Victor Hoover, Keith Armstrong, Lillian M. Stark, Geoffrey E. Hill, Thomas R. Unnasch

**Affiliations:** 1 Department of Botany and Plant Pathology, Oregon State University, Corvallis, Oregon, United States of America; 2 Department of Biological Sciences, Auburn University, Auburn, Alabama, United States of America; 3 Department of Global Health, College of Public Health, University of South Florida, Tampa, Florida, United States of America; 4 South Walton County Mosquito Control District, Santa Rosa Beach, Florida, United States of America; 5 North Walton County Mosquito Control District, DeFuniak Springs, Florida, United States of America; 6 Florida Department of Health, Bureau of Laboratories-Tampa, Tampa, Florida, United States of America; Blood Systems Research Institute, United States of America

## Abstract

For a variety of infectious diseases, the richness of the community of potential host species has emerged as an important factor in pathogen transmission, whereby a higher richness of host species is associated with a lowered disease risk. The proposed mechanism driving this pattern is an increased likelihood in species-rich communities that infectious individuals will encounter dead-end hosts. Mosquito-borne pathogen systems potentially are exceptions to such “dilution effects” because mosquitoes vary their rates of use of vertebrate host species as bloodmeal sources relative to host availabilities. Such preferences may violate basic assumptions underlying the hypothesis of a dilution effect in pathogen systems. Here, we describe development of a model to predict exposure risk of sentinel chickens to eastern equine encephalitis virus (EEEV) in Walton County, Florida between 2009 and 2010 using avian species richness as well as densities of individual host species potentially important to EEEV transmission as candidate predictor variables. We found the highest support for the model that included the density of northern cardinals, a highly preferred host of mosquito vectors of EEEV, as a predictor variable. The highest-ranking model also included *Culiseta melanura* abundance as a predictor variable. These results suggest that mosquito preferences for vertebrate hosts influence pathogen transmission.

## Introduction

Greater richness of host species has been associated with lowered risk of transmission in many studies of vector-borne pathogens (reviewed in [1], [2]). The mechanism underlying these associations may be either a lowering of competent host density that accompanies an increase in species richness [3], [4] or an increase in the proportion of “wasted” interactions – interactions of infectious individuals with noncompetent hosts [4]. Some researchers refer to the phenomenon of decreased pathogen transmission with greater species richness as a “dilution effect”, a concept closely related to that of zooprophylaxis [4]–[8]. Others reserve use of “dilution effect” strictly for lowered disease risk that accompanies increased species richness by means of a greater proportion of abortive interactions [4], [9].

Despite the evidence of dilution effects (in the broader sense), whether or not they are characteristic of all vector-borne diseases is currently a topic of debate in disease ecology [4], [8]. One argument against the ubiquity of dilution effects pertains to whether host reservoir competence is related to numerical dominance of the host. A dilution effect is expected for a pathogen system if the loss of biodiversity is accompanied by the removal of species that are poor reservoir hosts, leaving a more concentrated pool of competent hosts [2]. However, empirical evidence of such a relationship between numerical dominance and reservoir competence is currently lacking [9].

Other arguments against the generality of dilution effects for vector-borne disease pertain to vector ecology. For example, increases in abortive transmission events that accompany increases in species richness may be offset by increases in vector abundances, and this factor was not considered in initial dilution effect models [4], [9]. High variability in vector preferences for individual host species may also invalidate assumptions that underlie the dilution effect relationship between species richness and disease risk, and this variability has been invoked as a possible explanation for failure to detect a dilution effect in one study of mosquito-borne pathogen transmission [10].

A better understanding of the ubiquity of dilution effects in vector-borne pathogen systems is important both to advance a better conceptual framework for disease ecology, and to develop better predictive models of disease risk at specific locations. Advances in remote sensing technologies over the past 20 years, combined with an increased sophistication of occupancy modeling approaches, have allowed for more accessible and accurate maps of vertebrate distributions [11]–[13]. This increased availability of vertebrate distribution maps provides researchers with greater opportunities for using vertebrate community attributes to develop models to predict vector-borne diseases. Greater knowledge of the pathogen systems for which dilution effects are expected would identify host species richness as a candidate predictor with strong support for inclusion in model development for some pathogen systems, and at the same time, highlight pathogen systems where other attributes of the vertebrate community serve as better predictors of disease risk.

Alternative variables that may be derived from vertebrate distribution maps and may be potentially useful for predicting disease risk in lieu of host species richness are abundances of focal host species implicated in transmission. For example, in a study of transmission of West Nile virus (WNV; a pathogen that typically cycles between birds and mosquitoes) that found little support for a dilution effect, densities of focal host species were the primary factor identified as influencing disease risk [14]. The focal avian host species that were found to be influential were species assumed to be “high amplification hosts”, given field estimates suggesting that they had high amplification fractions (*F_i_*) relative to other species. Amplification fraction (*F_i_*) is calculated for host species *i* as the product of its relative abundance (*B_i_*), its selection index, i.e., its rate of use by mosquitoes as a bloodmeal source controlled for its availability relative to other host species (*P_i_*), and its reservoir competence (*C_i_*), i.e. F_i_ = B_i_*P_i_*C_i_ [15]. Despite its practical implications, the extent to which host species serve as better predictor of disease risk than host species richness in the specific cases of vector-borne pathogens wherein mosquitoes serves as the vectors is relatively unknown.

Here, we report the development of a spatially-explicit model for risk of exposure to eastern equine encephalomyelitis virus (EEEV) in sentinel chickens between 2009 and 2010 in Walton County, Florida. EEEV is among the rarest of the North American encephalitides but is of public health concern in the United States because of the risk it poses to humans and horses [16]. EEEV has a complex life cycle that involves multiple vertebrate reservoir hosts, primarily avian species, and vector mosquitoes [17], [18]. As such, avian species richness could play an important role in transmission of the virus. Our goals in this analysis were to determine the relative strength of evidence for an influence of avian species richness with risk of EEEV exposure in the southeastern United States compared to abundance of potentially influential individual host species and to develop a simple predictive model of EEEV exposure risk.

Our analysis focused specifically on EEEV exposure risk in sentinel chickens (*Gallus domesticus*), which are routinely used in surveillance for EEEV and play an important role in Early Warning Systems. Seroconversions of sentinel chickens to EEEV antibodies are associated with of EEEV infections in humans [19], [20]. Thus, a model predicting rates of EEEV exposure risk in chickens has the potential to be an important tool for predicting eastern equine encephalitis (EEE) risk to humans and horses at locations over broad geographic areas.

We used a multi-model inference approach to develop models of EEEV exposure risk in sentinel chickens during 2009 and 2010. Our decision to use multi-model inference reflects a paradigm shift currently underway in the fields of ecology and evolution (among others) away from null-hypothesis testing towards an analytical approach that considers multiple, competing hypotheses represented by statistical models [21]–[24]. We used a narrow set of plausible predictor variables in model development of EEEV exposure risk in sentinel chickens in an effort to minimize the risk of an unimportant variable occurring in our final selected model given our limited sample size [22].

We first considered the potential influence of avian species richness on EEEV exposure risk in sentinel chickens. To predict a dilution effect of avian species richness on EEEV transmission, we predicated our analysis on a set of simple assumptions. Following the classic understanding of EEEV transmission dynamics described by Scott and Weaver [18], we assumed that *Culiseta melanura* is the primary enzootic mosquito vector of the virus, that *Cs. melanura* is strictly ornithophilic and exhibits no host preferences when feeding on birds, and that only birds transmit EEEV to *Cs. melanura*. Recent research suggested deviation from some of these simple assumptions regarding the EEEV system [25]–[28], but these assumptions are broadly reasonable for the EEEV system and enable us to proceed with our efforts to predict virus transmission.

We next considered the influence of individual avian host species in model development. We used the densities of European starlings (*Sturnus vulgaris*) and northern cardinals (*Cardinalis cardinalis*) as candidate predictor variables in development of a model of EEEV exposure risk in chickens because both of these species potentially serve as high amplification hosts for EEEV. Data on the reservoir competences and selection indices of avian host species are available from only two studies [28], [29], and are limited to in the number of species for which such estimates exist. Nonetheless, northern cardinals and European starlings stand out as potential high amplification hosts. European starling is an invasive species in North America that has been shown to have the highest reservoir competence amongst all species tested in experimental inoculations with the virus [29]. We unfortunately do not have estimates of the selection index for this species. Thus, this species was considered to be potential high amplification host on the basis of its high reservoir competence alone. Similarly, we identified northern cardinal *Cardinalis cardinalis*, the second avian host species whose densities we considered in model development, on the basis of only one characteristic: its selection index. Northern cardinal was the only species present on study plots in the current study that was also identified as highly preferred host species of *Culiseta melanura* in a previous study [28]. Thus, we included northern cardinal density in model development given its strong likelihood of being a high amplification host because if its high attractiveness to *Culiseta melanura*.

In addition to contributions of avian community components, we also considered the influence of EEEV vector abundances on EEEV exposure risk in sentinel chickens. Specifically, we considered the influence of the abundance of *Culiseta melanura*–the putative primary enzootic vector of EEEV in North America –in model development. Because of their ornithophilic feeding habits and vector competences for the virus, *Culex restuans*, *Culex nigripalpus*, and *Culex erraticus*, have recently been proposed to influence enzootic transmission of EEEV, in addition to *Cs. melanura* [25], [26], [30], [31]. However, these proposed mosquito vectors all have lower vector competences than *Cs. melanura* for EEEV [30] and act more as generalists in their vertebrate feeding patterns than *Cs. melanura*, which feeds almost exclusively on birds [27], [28], [32]. As such, we expected that a unit change in the abundance of *Cs. melanura* would exert the greatest net change on EEEV exposure risk in birds, such that it was the candidate mosquito species with the most support for inclusion in model development.

## Materials and Methods

### Ethics Statement

Care of chickens at sentinel sites followed husbandry guidelines detailed by the Florida Department of Health [33]. These chickens are used for public health surveillance activities and are maintained by the county mosquito control districts. Blood was drawn from these chickens weekly as part of routine surveillance activities and not specifically for this study. Sentinel chicken monitoring conducted by these districts does not qualify as research, testing, or experimentation and thus does not require ethics committee approval [34]. We acquired permission from landowners to access sites for avian point-count surveys on private property.

### Data Collection

**Figure 1:**
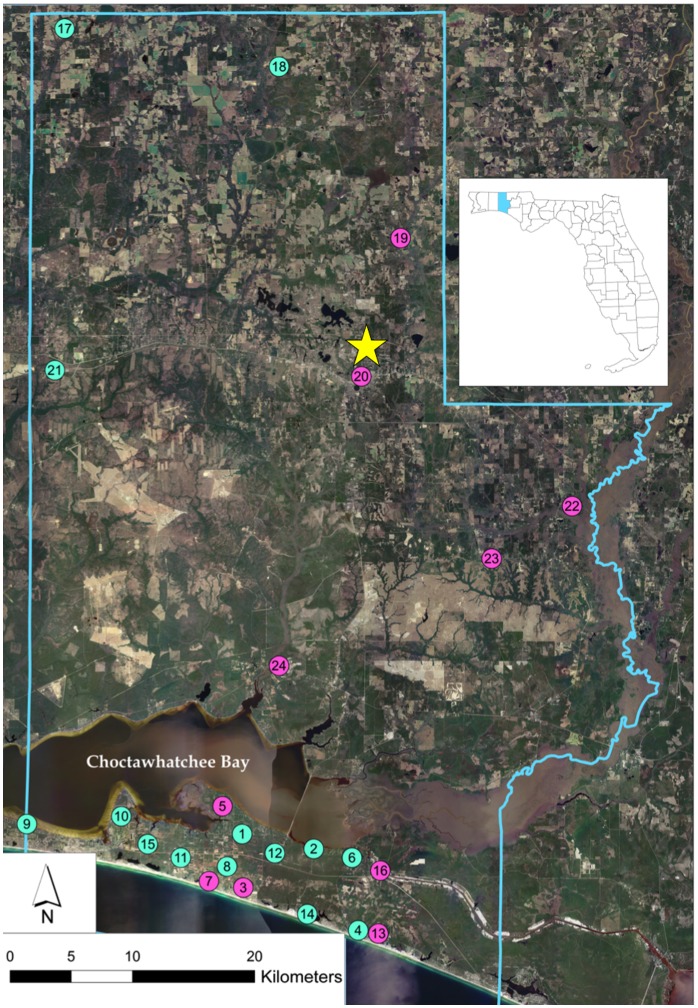
Walton County, Florida. Circles represent sentinel traps locations, where turquoise represents sites where EEEV exposure risk in sentinel chickens ≤0.010 seroconversions/chicken-week (median seroconversion incidence rate) and pink represents sites where EEEV exposure risk >0.010 seroconversions/chicken-week. Yellow star shows location of DeFuniak Springs, the Walton County seat. Subsetted image shows the location of Walton County within the state of Florida.

We collected data on the frequency of EEEV seroconversions in sentinel chickens as part of the North and South Walton County Mosquito Control Districts arbovirus surveillance programs. In total, we monitored 26 sentinel chicken flocks in 2009 and 2010 (Figure 1). Sentinel flocks were originally established as part of a statewide program for monitoring of St. Louis encephalitis virus (SLEV) in Florida in the late 1970s [19]. Although sentinel flock locations were chosen to optimize the success of that particular monitoring program, sentinel flocks are now used for routine surveillance for a range of arbovirus including EEEV and WNV. Blood samples drawn weekly from sentinel chickens were tested for the presence of EEEV neutralizing antibodies via hemaglutinnin inhibition and serum neutralization assays [35]. EEEV-positive chickens were removed from sentinel flocks following a positive test result and replaced with naïve chickens. The number of chickens monitored at sentinel sites varied between 2 and 6 (mean = 3.75, median = 3), with number of chickens monitored at individual sentinel sites held constant over the course of this study (Table S1). Constraints on the size of sentinel flocks included the number of chickens that can be successfully monitored by one full-time technician and the size of cages permitted on private properties.

**Figure 2:**
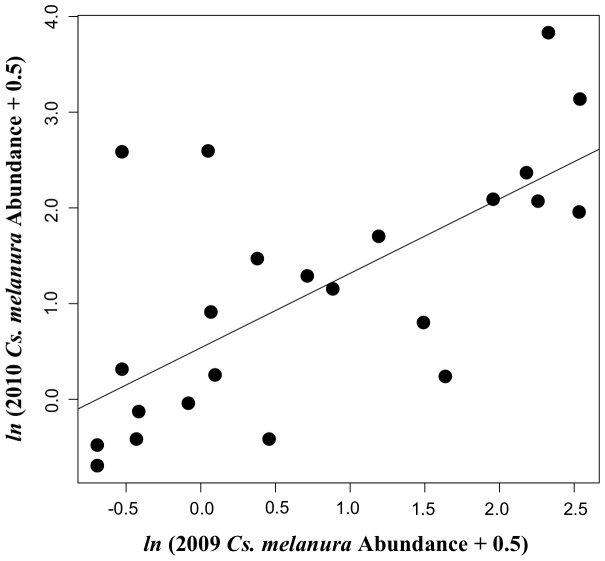
Association between annual *Culiseta melanura* abundances. Scatterplot showing the relationship between *Cs. melanura* abundance from April to October 2009 at 24 sentinel sites in Walton County, Florida with *Cs. melanura* abundance from the same sentinel sites and the same sampling period during 2010. Abundances from the two years are highly correlated (Spearman Rank Test, r_S_ = 0.63, p = 0.001). The best-fit line from simple linear regression is overlaid.

We quantified *Cs. melanura* abundance at the sentinel sites. These data originated from overnight collections of mosquitoes from New Jersey light traps and CDC light traps baited with CO_2_ located directly adjacent to each sentinel site location between April and October of 2009 and 2010. Collected mosquitoes were stored on wet ice for transport to district laboratories and were then identified using standard morphological keys [36]. We used the average number of *Cs. melanura* collected during the April-October period in 2009 and 2010 at each trap as the *Cs. melanura* abundance candidate predictor variable in model development described below. *Culiseta melanura* abundance averages across both 2009 and 2010 were used as overall abundance estimates because there was no evidence to suggest that the ranking of sites in order of *Cs. melanura* abundances differed between these years (Spearman Rank Test, r_S_ = 0.56, p = 0.004; Figure 2).

We quantified the avian communities surrounding sentinel sites using point-count surveys [37]. These avian surveys were conducted at 96 sites, with quartets of avian survey sites clustered around sentinel chicken flock sites. Specifically, individual avian survey sites in the quartet surrounding each sentinel flock were located at regular intervals along the perimeter of a buffer zone with radius-length 250 meters centered on the flock and the angular offset from north of each buffer randomized between 0 and 90 degrees. Point counts were not conducted directly next to sentinel cages due to interference from the chickens. A single observer trained in the vocal and visual identification of avian species that breed in southeastern United States visited survey sites between 0500 and 1000 EDT of June 2010. Each visit was divided into five 3-minutes during which species identification of all birds seen or heard within 100 m of the observer were recorded. We used this sampling protocol to keep our avian survey results consistent with the sampling scheme of a larger study of avian habitat associations, the results of which will be reported elsewhere. For this study, we estimated avian species richness at a sentinel site as the sum across all four surrounding survey sites and all 3-minute sessions, with species that were detected more than once only represented in counts once. We used the average densities across the four surrounding survey sites to estimate European starling and northern cardinal densities at each sentinel site.

### Model Development

We conducted our analysis in a multi-model inference framework [21]–[24], specifying a candidate set of general linear models for EEEV exposure risk in the sentinel chickens. Our measure of EEEV exposure risk was EEEV seroconversion incidence rate in the chickens: the ratio of the number of chickens that seroconverted at a site over the two-year study period to the chicken-time at risk, i.e. the product of number of chickens monitored at any one time at the site and 104 weeks. Mosquito and avian survey data were available for 24 of the original 26 sentinel sites monitored, such that we used this subset of the full set of 26 sentinel sites in model development.

The candidate model for seroconversion incidence rate in each year set consisted of fifteen models representing all possible combinations of four candidate predictor variables: avian species richness, European starling density, northern cardinal density, and average *Cs. melanura* abundance (2009–2010). We additionally included an intercept-only model in the candidate model set, whereby the mean response was modeled as constant across all sentinel sites. All predictor variables were standardized to have zero-means and standard deviations equal to unity.

We weighted candidate models by the difference in their bias-corrected Akaike Information Criterion (AICc, [38]) from that of the top-ranked model, i.e. the one with the lowest AICc. We based inference on weights assigned to individual models in the candidate set, and exclusion of zero in the 95% unconditional confidence intervals [UCIs] for coefficients of predictor variables, as averaged over all models in the candidate set. Models with AICc values within two units of the AICc of the top-ranked model were considered to be models with strong support [22]. Variables with coefficient 95% UCIs that excluded zero were inferred to be useful for prediction of EEEV exposure risk [22].

## Results

A total of 68 chickens seroconverted from a status of naive to positive for EEEV antibodies in 2009 across the 24 sites used in our analyses; 48 seroconverted in 2010 (Table S1). The average EEEV seroconversion incidence rate observed across all 24 sentinel sites was 0.013 seroconversions/chicken-week (median = 0.010, min = 0.000, max = 0.058) in 2009. Incidence rates of seroconversions in chickens during 2010 averaged 0.009 seroconversions/chicken-week (median = 0.008, min = 0.000, max = 0.032).

*Culiseta melanura* was present at 21 of the 24 sentinel sites for which mosquito data were available for the period of April-October of 2009 and was present at 22 of these sites in 2010. Average mosquito abundance across all sites was 3.23 individuals/trap-night (median = 1.02, min = 0.00, max = 12.16) in 2009 and 6.15 individuals/trap-night (median = 2.33, min = 0.00, max = 45.59).in 2010 (Table S2).

Individuals from 60 avian species were detected during point-count surveys, 38 of which were passerine species. Average avian species richness across all 24 sentinel sites was 22.83 (median = 21, min = 14, max = 41). The most common species detected were mourning dove *Zenaida macroura* and northern cardinal *Cardinalis cardinalis*, both of which occurred at all sites (Table S3). Blue jay *Cyanocitta cristata*, and Carolina wren *Thryothourus ludovivianus* occurred at all but one of the sentinel sites, and northern mockingbird *Mimus polyglottis* occurred at all but two. The rarest species, each detected at only one site, were black vulture *Cyoragyps atratus*, cliff swallow *Petrochelidon pyrrhonota*, house sparrow *Passer domesticus*, northern flicker *Colaptes auratus*, yellow-breasted chat *Icteria virens*, and yellow-throated vireo *Vireo flavifrons.* European starling occurred at 16 sites (Table S2). European starling density averaged 2.19 birds/km^2^ (median = 1.59, min = 0.00, max = 9.55) and the mean density of northern cardinal across all sentinel sites was 26.79 birds/km^2^ (median = 24.67, min = 6.37, max = 65.25).

**Figure 3:**
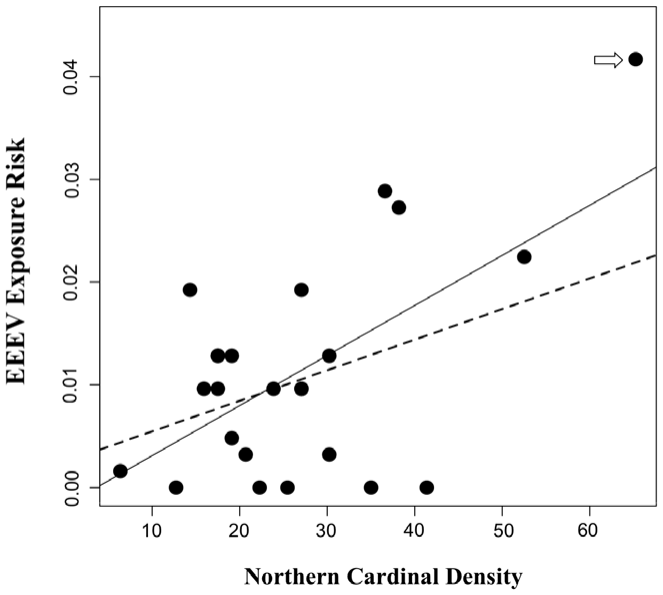
Association between EEEV exposure and northern cardinal density. Scatterplot showing the relationship between EEEV exposure risk in chickens during 2009 and 2010 and northern cardinal density at 24 sentinel sites in Walton County, Florida. The estimate for the slope of exposure risk regressed on northern cardinal density was 0.006 with a 95% UCI of [0.0025, 0.0107]. This estimated slope, when an influential observation (indicated by the arrow) was removed from the dataset, was 0.004 [−0.001, 0.009]. The best-fit line from simple linear regression of exposure risk residuals on northern cardinal density are overlaid, with the solid line fit to the full dataset, and the dashed line fit to the dataset that excluded the influential observation.

**Figure 4:**
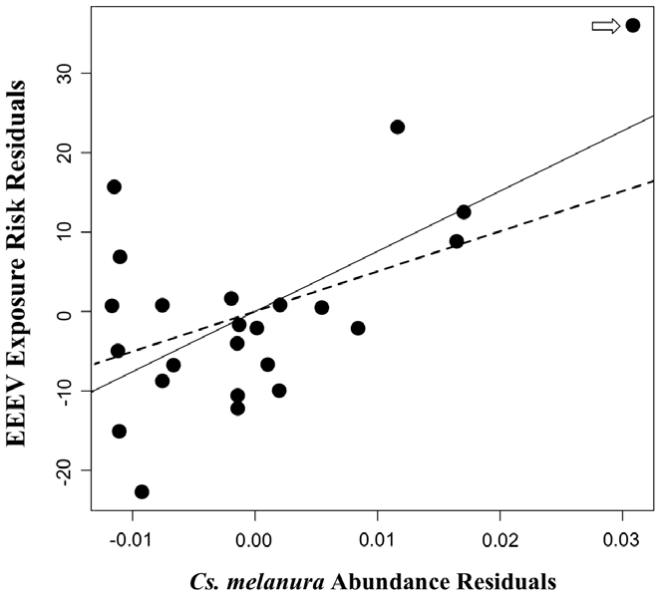
Association between EEEV exposure and *Cs. melanura* abundance. Added-variable plot showing the relationship between EEEV exposure risk in chickens during 2009 and 2010 and *Culiseta melanura* abundance at 24 sentinel sites in Walton County, Florida. The estimate for the slope of EEEV exposure risk regressed on *Cs. melanura* was 0.0028 with a 95% UCI of (−0.0012, 0.0058). *Cs. melanura* abundance residuals = residuals from regression of northern cardinal density on *Cs. melanura* abundance, EEEV exposure risk residuals = residuals from regression of EEEV exposure risk residuals on *Cs. melanura* abundance. The best-fit line from simple linear regression of EEEV exposure risk residuals on northern cardinal density residuals are overlaid, with the solid line fit to the full dataset, and the dashed line fit to the dataset that excluded the influential observation shown in Figure 3.

The model of EEEV exposure risk in sentinel chickens that had the strongest support included one predictor: northern cardinal density (Figure 3). One other model had strong support, i.e. ΔAICc <2; it included northern cardinal density and *Cs. melanura* abundance as predictor variables (Table 1, Figure 4).

**Table 1:** Attributes of highest-ranking models in candidate set used in spatial modeling of EEEV exposure risk in sentinel chickens in Walton County, Florida in 2009 and 2010.

Model	log(*L*)	AICc	K	Δ_i_	w_i_
NOCA	80.52	−153.84	3	0.00	0.36
mel+NOCA	81.66	−153.21	4	0.63	0.26
avian+NOCA	80.64	−151.17	4	2.67	0.09
EUST+NOCA	80.59	−151.07	4	2.77	0.09
avian+mel+NOCA	82.08	−150.82	5	3.02	0.08
EUST+mel+NOCA	81.66	−149.99	5	3.86	0.05
avian+EUST+NOCA	80.75	−148.18	5	5.67	0.02
avian+EUST+mel+NOCA	82.10	−147.26	6	6.59	0.01
avian	76.83	−146.46	3	7.39	0.01
null	75.22	−145.87	2	7.97	0.01
avian+EUST	77.72	−145.34	4	8.50	0.01
EUST	75.93	−144.65	3	9.19	0.00
avian+mel	77.37	−144.63	4	9.21	0.00
mel	75.27	−143.35	3	10.50	0.00
avian+EUST+mel	78.15	−142.98	5	10.87	0.00
EUST+mel	75.94	−141.78	4	12.06	0.00
NOCA	78.34	−149.41	3	0.00	0.18
null	76.76	−148.92	2	0.49	0.14
mel+NOCA	79.47	−148.72	4	0.69	0.13
avian	77.76	−148.26	3	1.15	0.10
avian+mel	78.92	−147.62	4	1.79	0.07
mel	77.18	−147.10	3	2.31	0.06
avian+NOCA	78.61	−147.00	4	2.41	0.05
EUST	77.08	−146.89	3	2.52	0.05
avian+mel+NOCA	80.19	−146.85	5	2.56	0.05
EUST+NOCA	78.42	−146.61	4	2.80	0.04
avian+EUST	78.21	−146.20	4	3.21	0.04
EUST+mel+NOCA	79.48	−145.42	5	3.99	0.02
avian+EUST+mel	79.24	−144.95	5	4.46	0.02
EUST+mel	77.41	−144.60	4	4.81	0.02
avian+EUST+NOCA	78.78	−144.04	5	5.37	0.01
avian+EUST+mel+NOCA	80.23	−143.22	6	6.19	0.01

Models described below the dotted line are those that were developed using the dataset that excluded an influential observation.

AICc = bias-corrected Akaike Information Criterion.

K = no. parameters estimated.

Δ_i_ = difference in AICc from the model that minimized the AICc.

w_i_ = AICc weight.

Variable names: mel = Cs. melanura abundance, avian = avian species richness, EUST = European starling density, NOCA = northern cardinal density.

The model-averaged estimate for the coefficient of northern cardinal density in the EEEV exposure risk model was positive (0.007) with an unconditional standard error (USE) = 0.002. We inferred this variable to be useful for predictive model development given exclusion of zero from its 95% UCI: [0.0025,0.0107]. The coefficient estimate for *Cs. melanura* abundance was also positive: 0.0028 (USE = 0.0020), as was the coefficient estimate for avian species richness: 0.0016 (USE = 0.0024). The coefficient estimate for European starling density was negative: −0.001 (USE = 0.0022); however, we inferred neither *Cs. melanura* abundance, avian species richness, nor European starling density to be useful for prediction, given that their estimated coefficient 95% UCIs included zero: [−0.0012, 0.0068], [−0.0032, 0.0063], [−0.0049, 0.0037], respectively (Table 2).

**Table 2:** Importance weights and results of model averaging for predictor variables in spatial modeling of EEEV exposure risk in sentinel chickens in Walton County, Florida in 2009 and 2010.

	Model-averaged	Weighted	95% Confidence Interval		Importance
Variable	Estimate	Unconditional SE	Lower	Upper	Weight
Intercept	0.0113	0.0019	0.0075	0.0150	1.00
NOCA	0.0066	0.0021	0.0025	0.0107	0.97
mel	0.0028	0.0020	−0.0012	0.0068	0.41
avian	0.0016	0.0024	−0.0032	0.0063	0.23
EUST	−0.0006	0.0022	−0.0049	0.0037	0.19
Intercept	0.0102	0.0019	0.0065	0.0140	1.00
NOCA	0.0040	0.0025	−0.0010	0.0090	0.50
mel	0.0025	0.0020	−0.0015	0.0064	0.38
avian	0.0025	0.0022	−0.0017	0.0068	0.36
EUST	−0.0011	0.0021	−0.0051	0.0029	0.21

Results below the dotted line are based on the development of models using the dataset that excluded an influential observation.

Variable names: mel = Culiseta melanura abundance, avian = avian species richness, EUST = European starling density, NOCA = northern cardinal density.

Results of diagnostic tests of the final predictive model of 2009–2010 EEEV exposure risk in sentinel chickens, i.e. that which included northern cardinal density as a predictive variable, indicated general compliance with standard regression assumptions [39]: We detected no discernible patterns in residual plots. The null hypothesis of homoscedasticity of error variance was not rejected using the Breusch-Pagan test (BP = 3.83, df = 1, p>0.05). The deviance residuals of this model were not spatially autocorrelated (*Moran’s I* = −0.03, p>0.05). However, assessment of the influence of points on regression coefficients estimates and model fitted values using the criteria of and DFFITS, Cook’s distance, and DFBETAS measures revealed one potentially influential point. The first two of these measures assesses the influence of an observation on its fitted values and overall model fit, respectively, and DFBETAS assesses the influence of observations on coefficient estimates [39]. The point that was inferred to be highly influential based on these diagnostics was from a site where chickens had the highest EEEV exposure risk. This point had a DFFITS value of 1.51 and a DFBETAS value of 1.43, both of which are above the standard cutoff criterion of 1for small datasets, and a Cook’s distance value of 1.03, which also exceeds the criterion for classification as an influential observation [39].

There was no reason to discard the influential observation from the analysis despite its potentially high influence, as no errors in data entry were associated with this point upon further checking. Nonetheless, we repeated model development excluding this point from the dataset, as is recommended in such circumstances [39], towards the goal of a thorough presentation of modeling results. Inference based on model selection and parameter estimates using this reduced dataset were similar to that using the full dataset, whereby the top-ranked model included northern cardinal as the sole predictor variable (Table 1). None of the predictor variables considered in model development were inferred to be useful for prediction of EEEV exposure rates using this reduced dataset. However, signs of the coefficients of variables, and well as the rank of variables in terms of their importance weights were also consistent with the results of analysis of the full dataset (Table 2).

## Discussion

Vertebrates play integral roles in the transmission of vector-borne pathogens either enhancing or reducing pathogen cycling in natural communities [40]. In recent research on the role of vertebrate hosts in vector-borne disease, two competing hypotheses have emerged. The dilution effect hypothesis proposes that the entire community of vertebrate hosts collectively shapes disease risk and thus that indices of species diversity can be used to characterize disease risk [5]–[7]. The alternative hypothesis is that the abundance of individual species, or subsets of species, of the vertebrate host community determine disease risk [14]. Our analysis of these two competing hypotheses in a mosquito-borne pathogen system revealed strong support for the latter. We found evidence that the abundance of one preferred host of a primary vector of EEEV, the northern cardinal, determined pathogen exposure risk in sentinel chickens in the EEEV system.

A strong influence of specific vertebrate hosts has also been found in studies of transmission of West Nile virus, another mosquito-borne pathogen system. The American robin (*Turdus migratorius*) is the species most frequently identified as contributing to transmission of West Nile virus both because it is a highly preferred host and has relatively high reservoir competence [41]–[44]. However, other studies of this system have also found strong evidence for dilution effects, whereby increasing avian species richness is associated with reduced disease risk [45]–[47]. Thus, more studies are needed to determine whether the effect on of a single dominant species on EEEV pathogen transmission is stronger than the total composition of the avian community such as we found in this study.

Although our analysis revealed that the risk of EEEV exposure in sentinel chickens was strongly affected by the abundance of northern cardinals, the exact mechanism that underlies this association is still unclear. We based our hypothesis of a potential influence of northern cardinal abundance on two observations: 1) cardinals would qualify as a “high amplification” species in communities with uniform relative abundances of avian hosts and 2) the abundances of host species that met this same criterion in studies of WNV were associated with disease risk [15]. Species with high amplification fractions are the same species that would be expected to have the greatest change in the relative R_0_ (R_0, rel_), the increase in the pathogen reproductive ratio due to heterogeneity in vector feeding and reservoir competences of hosts, associated with changes in its relative abundance [41]. This pattern emerges because the amplification fraction ultimately reduces to F_i_ = B_i_^2^*C_i_, whereby *C_i_* is host reservoir competence and *B_i_* is the proportion of bloodmeals derived from host species *i* [42]. For each host species, B_i_^2^*C_i_ is divided by its relative abundance to determine its individual contribution to (R_0, rel_), such that species with large values for this product (i.e. larger amplification fractions), will make greater contributions to (R_0, rel_) per unit of relative abundance than those with small amplification fractions. In the case of WNV, R_0, rel_ is associated with pathogen transmission at multiple study sites [41]. Thus, by extension, the relative abundances of species with large amplification fractions may contribute the most to variation in pathogen transmission by dominating spatial variability in R_0, rel_.

The relative abundance of northern cardinals was strongly associated with northern cardinal density in this study (post-hoc analysis: r_S_ = 0.52, p<0.001), so the observed association between northern cardinal density and EEEV exposure risk is confounded by cardinal abundance. Additionally, although the reservoir competence of northern cardinal in a laboratory study was above average compared to other species tested, this species differed more in terms of attractiveness to *Cs. melanura* compared to other host species than it did terms of reservoir competence based on percentile ranks (0.76 and 0.60, respectively) [28], [29]. The attractiveness of northern cardinals to EEEV vectors, rather than reservoir competence of northern cardinals, may be responsible for the association between northern cardinal density and EEEV exposure risk in sentinel chickens.

Identification of the mechanism driving the positive relationship we observed between northern cardinal density and EEEV exposure risk clearly warrants further research. Our results suggest that northern cardinal density may be useful for predicting EEEV exposure risk among sentinel chickens in Florida. Florida has reported the highest number of human and equine cases of EEEV in the North America over the past half-century [48]. If the rate of exposure in sentinel chickens covaries with the rate of exposure in humans and other mammals, then models such as ours, based on assessment of bird communities, could be used to make decisions about EEEV control measures within the state of Florida.

*Culiseta melanura* has long been hypothesized to be the primary enzootic vector of EEEV [14], [15], and our results lend support to this hypothesis. The abundance of *Cs. melanura* emerged as a predictor variable in one of the two models that had strong support. Additionally, *Cs. melanura* had the second highest importance weight of the four variables considered in model development. However, *Cs. melanura* abundance was not inferred as a variable useful for prediction of EEEV exposure rates in sentinel chickens. Thus, these observations suggest that the influence of *Cs. melanura* abundance on EEEV transmission has the potential to be obscured by variability in northern cardinal abundances in spatial analyses of EEEV disease risk. Additionally, an association between EEEV exposure risk and *Cs. melanura* abundance may have been obscured by measurement error present in the *Cs. melanura* abundance data due to the use of two different mosquito trap types used throughout the study area.

This study is based on a limited number of sampling sites, such that we considered only the most biologically reasonable variables for which data were available in model development. Unfortunately, however, the small sample size of this study precluded us from considering a wider range of variables that may influence EEEV transmission. For example, mosquito species other than *Cs. melanura* found in Walton County (detailed recently in [49]) may play a role in EEEV transmission [25], [26], [30], [31]. Supplementary evidence of high seroprevalences of EEEV in both *Cs.melanura* and northern cardinal populations in the study area relative to other species would also be useful to confirm results of this study. Also, the limited number of sentinel sites prohibited us from splitting the dataset to create a validation dataset. Nonetheless, this study provides important insight in supporting for a role of northern cardinal density in predicting EEEV exposure risk. Such insight will be important in informing model development in future studies investigating patterns of occurrence of EEE in humans and horses, and concurrently, provides evidence counter to a dilution effect in mosquito-borne pathogens systems.

## Supporting Information

Table S1 **EEEV exposures of sentinel chickens in Walton County, Florida between 2009 and 2010, as identified through seroconversions from EEEV-antibody negative statuses to positive statuses.**
(XLSX)

Table S2 **Estimates of** ***Culiseta melanura*** **abundances derived from mosquito sampling from April to mid-October during 2009 and 2010 in Walton County, Florida.**
(XLSX)

Table S3 **Occupancy matrix for avian species detected during avian surveys in June 2010 at sentinel chicken flock site locations in Walton County, Florida.**
(XLS)

## References

[1] Keesing F, Holt RD, Ostfeld RS (2006) Effects of species diversity on disease risk. Ecol Lett 9: 485–498. 16623733 10.1111/j.1461-0248.2006.00885.x

[2] Keesing F, Belden LK, Daszak P, Dobson A, Harvell CD, et al (2010) Impacts of biodiversity on the emergence and transmission of infectious diseases. Nature 468: 647–652. 21124449 10.1038/nature09575 PMC7094913

[3] Rudolf VHW, Antonovics J (2005) Species coexistence and pathogens with frequency-dependent transmission. Am Nat 166: 112–118. 15937794 10.1086/430674

[4] Begon M (2008) Effects of host diversity on disease dynamics. In Ostfeld RS, Keesing F, Eviner VT, editors. Infectious disease ecology: effects of ecosystems on disease and of disease on ecosystems. Princeton: Princeton University Press. 12–29.

[5] LoGiudice K, Ostfeld RS, Schmidt KA, Keesing F (2003) The ecology of infectious disease: Effects of host diversity and community composition on Lyme disease risk. Proc Natl Acad Sci USA 100: 567–571. 12525705 10.1073/pnas.0233733100 PMC141036

[6] Schmidt KA, Ostfeld RS (2001) Biodiversity and the dilution effect in disease ecology. Ecology 82: 609–619.

[7] Mitchell CA, Tilman D, Groth JV (2002) Effects of grassland plant species diversity, abundance, and composition on foliar fungal disease. Ecology 83: 1713–1726.

[8] Hess AD, Hayes RO (1970) Relative potentials of domestic animals for zooprophylaxis against mosquito vectors of encephalitis. Am J Trop Med Hyg 19: 327–334. 5443080 10.4269/ajtmh.1970.19.327

[9] Randolph SE, Dobson ADM (2012) Pangloss revisited: a critique of the dilution effect and the biodiversity-buffers-disease paradigm. Parasitology 139: 847–863. 22336330 10.1017/S0031182012000200

[10] Loss SR, Hamer GL, Walker ED, Ruiz MO, Goldberg TL, et al (2009) Avian host community structure and prevalence of West Nile virus in Chicago, Illinois. Oecologia 159: 415–424. 19034529 10.1007/s00442-008-1224-6

[11] Kushwaha SPS, Roy PS (2002) Geospatial technology for wildlife habitat evaluation. Trop Eco. 43: 137–150.

[12] Araujo MB, Guisan A (2006) Five (or so) challenges for species distribution modeling. J Biogeogr 33: 1677–1688.

[13] MacKenzie DI, Nichols JD, Royle JA, Pollock KH, Bailey LA, et al.. (2006) Occupancy modeling and estimation. San Diego: Elsevier. 344 p.

[14] McKenzie VJ, Goulet NE (2010) Bird community composition linked to human West Nile virus cases along the Colorado Front Range. EcoHealth 7: 439–447. 21125307 10.1007/s10393-010-0360-8

[15] Hamer GL, Kitron UD, Goldberg TL, Brawn JD, Loss SR, et al (2009) Host selection by *Culex pipiens* mosquitoes and West Nile virus amplification. Am J Trop Med Hyg 80: 268–278. 19190226

[16] Villari P, Spielman A, Komar N, McDowell M, Timperi RJ (1995) The economic burden imposed by a residual case of eastern encephalitis. Am J Trop Med Hyg 52: 8–13. 7856830 10.4269/ajtmh.1995.52.8

[17] Morris CD (1988) Eastern equine encephalomyelitis. In: Monath TP, editor. The Arboviruses: Epidemiology and Ecology. Boca Raton: CRC Press. 1–20.

[18] Scott TW, Weaver SC (1989) Eastern equine encephalomyelitis virus: Epidemiology and evolution of mosquito transmission. Adv Vir Res 37: 277–328. 10.1016/s0065-3527(08)60838-6 2574935

[19] Day JF (1989) The use of sentinel chickens for arbovirus surveillance in Florida. J Fla Anti-mosq Assoc 60: 58–61.

[20] Day JF, Stark LM (1996) Transmission patterns of St. Louis encephalitis and eastern equine encephalitis viruses in Florida: 1978–1993. J Med Entomol 33: 132–139. 8906917 10.1093/jmedent/33.1.132

[21] Anderson DR, Burnham KP, Thompson WL (2000) Null hypothesis testing: problems, prevalence, and an alternative. J Wildl Manage 64: 912–923.

[22] Burnham KP, Anderson DR (2002) Model selection and multi-model inference: A practical information-theoretic approach. New York: Springer-Verlag. 496 p.

[23] Richards SA, Whittingham MJ, Stephens PA (2010) Model selection and model averaging in behavioural ecology: the utility of the IT-AIC framework. Behav Ecol Sociobiol 65: 77–89.

[24] Grueber CE, Nakagawa S, Laws RJ, Jamieson IG (2011) Multimodel inference in ecology and evolution: challenges and solutions. J Evol Biol 24: 699–711. 21272107 10.1111/j.1420-9101.2010.02210.x

[25] Cupp EW, Klingler K, Hassan HK, Viguers LM, Unnasch TR (2003) Transmission of eastern equine encephalomyelitis virus in Central Alabama. Am J Trop Med Hyg 68: 495–500. 12875303 PMC2575747

[26] Cupp EW, Tennessen KJ, Oldland WK, Hassan HK, Hill GE, et al (2004) Mosquito and arbovirus activity during 1997–2002 in a wetland in northeastern Mississippi. J Med Entomol 41: 495–501. 15185956 10.1603/0022-2585-41.3.495 PMC2581464

[27] Molaei G, Oliver JA, Andreadis TG, Armstrong PM, Howard JJ (2006) Molecular identification of blood-meal sources in *Culiseta melanura* and *Culiseta morsitans* from an endemic focus of eastern equine encephalitis virus in New York. Am J Trop Med Hyg 75: 1140–1147. 17172382

[28] Estep LK, McClure CJW, Burkett-Cadena ND, Hassan HK, Hicks TL, et al (2011) A multi-year study of mosquito feeding patterns on avian hosts in a southeastern focus of eastern equine encephalitis virus. Am J Trop Med Hyg 84: 718–726. 21540380 10.4269/ajtmh.2011.10-0586 PMC3083738

[29] Komar N, Dohm DJ, Turell MJ, Spielman A (1999) Eastern equine encephalitis virus in birds: Relative competence of European starlings (*Sturnus vulgaris*). Am J Trop Med Hyg 60: 387–391. 10466964 10.4269/ajtmh.1999.60.387

[30] Chamberlain RW, Sikes RK, Nelson DB, Sudia WD (1954) Studies on the North American arthropod-borne encephalitides: VI. Quantitative determinants of virus-vector relationships. Am J Hyg 60: 278–285. 13207099 10.1093/oxfordjournals.aje.a119721

[31] Cohen SB, Lewoczko K, Huddleston DB, Moody E, Mukherjee S, et al (2009) Host feeding patterns of potential vectors of eastern equine encephalitis virus at an epizootic focus in Tennessee. Am J Trop Med Hyg 81: 452–456. 19706914

[32] Edman JD, Webber LA, Kale HWII (1972) Host-feeding patterns of Florida mosquitoes. II. *Culiseta* . J Med Entomol 9: 429–434. 4404140 10.1093/jmedent/9.5.429

[33] Florida Department of Health (2012) Surveillance and control of selected mosquito-borne diseases in Florida. http://www.doh.state.fl.us/Environment/medicine/arboviral/index.html.

[34] Animal and Plant Health Inspection Service, United States Department of Agriculture (2009) “Animals and Animal Products.” 9 CFR 1.1 and 2.30. Available: http://www.gpo.gov/fdsys/pkg/CFR-2009-title9-vol1/xml/CFR-2009-title9-vol1-chapI-subchapA.xml.

[35] Smith JP, Tennant RA, Kozak JA III, Cope EH, Walsh JD (2008) Trapping and serosurveillance of live wild birds for arboviruses. Tech Bull Fla Mosq Control Assoc 8: 10–14.

[36] Darsie RF Jr, Ward RA (2005) Identification and geographical distribution of the mosquitoes of North America, north of Mexico. Gainesville: University Press of Florida. 400 p.

[37] Ralph JC, Droege S, Sauer JR (1995) Managing and monitoring birds using point counts: standards and applications. In: Ralph JC, Sauer JR, Droege S, editors. Monitoring bird populations by point counts: U.S. Forest Service General Technical Report PSWGTR-149. Albany, CA: USDA Forest Service, Pacific Southwest Research Station. 161–168.

[38] Hurvich CM, Tsai CL (1989) Regression and time series model selection in small samples. Biometrika 76: 297–307.

[39] Kutner MH, Nachtsheim CJ, Neter J, Li W (2004) Applied linear statistical models, 5^th^ edn. New York,: Mcgraw-Hill/Irwin. 1396 p.

[40] Chaves LF, Hernandez MJ (2004) Mathematical modeling of American cutaneous leishmaniasis: incidental hosts and threshold conditions for infection persistence. Acta Trop 92: 245–252. 15533294 10.1016/j.actatropica.2004.08.004

[41] Kilpatrick AM, Daszak P, Jones MJ, Marra PP, Kramer LD (2006) Host heterogeneity dominates West Nile virus transmission. Proc R Soc B Biol Sci 273: 2327–2333. 10.1098/rspb.2006.3575 PMC1636093 16928635

[42] Kent R, Juliusson L, Weissmann M, Evans S, Komar N (2009) Seasonal Blood-Feeding Behavior of *Culex tarsalis* (Diptera: Culicidae) in Weld County, Colorado, 2007. J Med Entomol 46: 380–390. 19351092 10.1603/033.046.0226

[43] Diuk-Wasser MA, Molaei G, Simpson JE, Folsom-O’Keefe CM, Armstrong PA, et al (2010) Avian communal roosts as amplification foci for West Nile virus in urban areas in northeastern United States. Am J Trop Med Hyg 82: 337–343. 20134014 10.4269/ajtmh.2010.09-0506 PMC2813178

[44] Simpson JE, Hurtado PJ, Medlock J, Molaei G, Andreadis TG, et al (2011) Vector host-feeding preferences drive transmission of multi-host pathogens: West Nile virus as a model system. Proc. R. Soc. B 279: 925–933. 10.1098/rspb.2011.1282 PMC3259921 21849315

[45] Ezenwa V, Godsey M, King R, Guptill S (2006) Avian diversity and West Nile virus: testing associations between biodiversity and infectious disease risk. Proc R Soc B 273: 109–117. 10.1098/rspb.2005.3284 PMC1560012 16519242

[46] Swaddle JP, Calos SE (2008) Increased avian diversity is associated with lower incidence of human West Nile infection: Observation of the dilution effect (2008) PLoS One. 3: e2488. 10.1371/journal.pone.0002488 PMC2427181 18575599

[47] Allan BF, Langerhans RB, Ryberg WA, Landesman WJ, Griffin NW, et al (2009) Ecological correlates of risk and incidence of West Nile virus in the United States. Oecologia 158: 699–708. 18941794 10.1007/s00442-008-1169-9

[48] Eastern Equine Encephalitis, U.S. Department of Health and Human Services, Centers for Disease Control and Prevention (2012) Eastern Equine Encephalitis: Epidemiology & Geographic Distribution. Available: http://www.cdc.gov/easternequineencephalitis/tech/epi.html. Accessed 2012 Feb 2.

[49] Vander Kelen PT, Downs JA, Burkett-Cadena NB, Ottendorfer CL, Hill K, et al (2012) Habitat associations of eastern equine encephalitis transmission In Walton County Florida. J Med Entomol 49: 746–756. 22679885 10.1603/me11224 PMC3552394

